# Draft Genome Sequence of Paenalcaligenes niemegkensis NGK35^T^, Isolated from Plastic-Polluted Soil of an Abandoned Landfill

**DOI:** 10.1128/mra.00671-22

**Published:** 2022-08-31

**Authors:** Alexander Bartholomäus, Julia Mitzscherling, Daniel Lipus, Joana MacLean, Dirk Wagner

**Affiliations:** a GFZ German Research Centre for Geosciences, Section Geomicrobiology, Potsdam, Germany; b University of Potsdam, Institute of Geoscience, Potsdam, Germany; Wellesley College

## Abstract

The Gram-negative bacterium Paenalcaligenes niemegkensis NGK35^T^ was isolated from plastic debris in an abandoned landfill. It has the ability to grow on polyethylene and hexadecane as the sole carbon sources. Here, we report the corresponding draft genome, which contains 3.66 Mbp and is characterized by a G+C content of 52.1%.

## ANNOUNCEMENT

Paenalcaligenes niemegkensis NGK35^T^ was recently isolated in the frame of a study targeting potential polyethylene-degrading microorganisms and was described as a potential new member of the genus *Paenalcaligenes*, which includes three established species to date. The recently described Gram-negative bacterial strain (1) was isolated from plastic-polluted soil of an abandoned landfill in the eastern part of Germany (52°02′58.8″N, 12°39′34.8″E), which thus adds to the variety of environments in which this genus can be found. Other *Paenalcaligenes* members were isolated from human blood (2), from spent mushroom compost (3), or from the larval gut of the black soldier fly, Hermetia illucens (4).

Plastic microbial isolates were enriched in slurries with plastic debris from the landfill and minimal salt medium (MSM) containing cycloheximide (1% of a 1 M solution) but no additional carbon source, according to a protocol described by Burd (5). After 3 to 5 days at 27°C, slurries were plated on solid MSM containing 0.1% powdered and UV-weathered polyethylene as an additional carbon source. Single colonies were transferred from polyethylene-containing MSM to MSM with 1% *n*-hexadecane as the only carbon source. Genomic DNA of strain NGK35^T^ was extracted from cell material resuspended in extraction buffer using the UltraClean microbial DNA isolation kit (MoBio, Carlsbad, CA, USA).

High-molecular-weight DNA was prepared using a rapid barcoding sequencing kit (Oxford Nanopore Technologies [ONT], Oxford, UK) and cleaned up using AMPure XP beads (Beckman Coulter, Pasadena, CA, USA). Sequencing was performed using the MinION sequencing platform (ONT) and the FLO-MIN106 flow cell. The library was sequenced for 72 h, and the quality was monitored using the MinKNOW interface (ONT). Default parameters were used for all software unless otherwise specified. Raw sequencing data were base called and demultiplexed using high accuracy with guppy v4.4.2+9623c1626 (ONT), which resulted in 80,552 raw reads with an *N*_50_ value of 3,231 bp. Assembly and polishing were performed with Flye v2.8.2-b1689 (6) (parameters: –plasmid –meta). A draft genome quality assessment was conducted using CheckM v1.0.13 (7) with the lineage_wf workflow. Full-length 16S rRNA gene sequences were recovered using the CheckM ssu_finder tool (7). Draft genome characteristics were assessed using the Quality Assessment Tool for Genome Assemblies (QUAST) (8). Gene annotation of the draft genome was performed using the NCBI Prokaryotic Genome Annotation Pipeline (PGAP) (9).

The resulting draft genome had a size of 3,665,035 bp across 7 contigs, with a G+C content of 52.1%, an *N*_50_ value of 2,371,143 bp, and coverage of 62×. The genome was found to be 96.3% complete and 0.71% contaminated. The taxonomic affiliation was inferred using GTDB-Tk v1.5.0 (10), which suggested that the isolate belongs to the genus *Paenalcaligenes*. The recovered draft genome was compared to other available *Paenalcaligenes* genomes by calculating the average nucleotide identity (ANI) (ANIb based on BLAST+ and ANIm based on MUMmer) using the JSpeciesWS tool (11) (accessed 21 September 2021). Results suggested that the strain NGK35^T^ draft genome was most closely related to that of Paenalcaligenes suwonensis 191B (Genbank accession number JAANPV000000000) (ANIb, 70.27%; ANIm, 84.01%). A phylogenetic evaluation of the recovered full-length 16S rRNA gene sequence confirmed this observation, with 96.9% sequence identity to P. suwonensis ABC02-12^T^ (Genbank accession number NR_133804) across 1,543 bp determined using NCBI BLAST (12).

### Data availability.

The draft genome and gene annotation of Paenalcaligenes niemegkensis NGK35^T^ are available at NCBI with the GenBank accession number JAKGCT000000000 and the BioProject accession number PRJNA797906. The raw reads are available via the SRA accession number ERR7440776, and the 16S rRNA gene sequence is available via the GenBank accession number ON900080. The strain is available via the German Collection of Microorganisms and Cell Cultures with the accession number DSM 113270^T^ and the Netherlands Culture Collection of Bacteria with the accession number NCCB 100854^T^.
